# Polygenic risk for coronary heart disease acts through atherosclerosis in type 2 diabetes

**DOI:** 10.1186/s12933-020-0988-9

**Published:** 2020-01-30

**Authors:** Tianyuan Lu, Vincenzo Forgetta, Oriana H. Y. Yu, Lauren Mokry, Madeline Gregory, George Thanassoulis, Celia M. T. Greenwood, J. Brent Richards

**Affiliations:** 1 Centre for Clinical Epidemiology, Lady Davis Institute for Medical Research, Jewish General Hospital, Montreal, QC Canada; 2 Quantitative Life Sciences Program, McGill University, Montreal, QC Canada; 3 Division of Endocrinology, Jewish General Hospital, Montreal, QC Canada; 4 Department of Medicine, McGill University, Montreal, QC Canada; 5 Preventive and Genomic Cardiology, McGill University Health Centre and Research Institute, Montreal, QC Canada; 6 Department of Human Genetics, McGill University, Montreal, QC Canada; 7 Gerald Bronfman Department of Oncology, McGill University, Montreal, QC Canada; 8 Department of Epidemiology, Biostatistics & Occupational Health, McGill University, Montreal, QC Canada; 9 Department of Twin Research and Genetic Epidemiology, King’s College London, Strand, London, UK; 10 Jewish General Hospital, Room H-413, 3755 Côte Sainte-Catherine Road, Montreal, QC H3T 1E2 Canada

**Keywords:** Atherosclerosis, Coronary heart disease, Polygenic risk scores, Risk factors, Type 2 diabetes

## Abstract

**Background:**

Type 2 diabetes increases the risk of coronary heart disease (CHD), yet the mechanisms involved remain poorly described. Polygenic risk scores (PRS) provide an opportunity to understand risk factors since they reflect etiologic pathways from the entire genome. We therefore tested whether a PRS for CHD influenced risk of CHD in individuals with type 2 diabetes and which risk factors were associated with this PRS.

**Methods:**

We tested the association of a CHD PRS with CHD and its traditional clinical risk factors amongst individuals with type 2 diabetes in UK Biobank (N = 21,102). We next tested the association of the CHD PRS with atherosclerotic burden in a cohort of 352 genome-wide genotyped participants with type 2 diabetes who had undergone coronary angiograms.

**Results:**

In the UK Biobank we found that the CHD PRS was strongly associated with CHD amongst individuals with type 2 diabetes (OR per standard deviation increase = 1.50; p = 1.5 × 10^− 59^). But this CHD PRS was, at best, only weakly associated with traditional clinical risk factors, such as hypertension, hyperlipidemia, glycemic control, obesity and smoking. Conversely, in the angiographic cohort, the CHD PRS was strongly associated with multivessel stenosis (OR = 1.65; p = 4.9 × 10^− 4^) and increased number of major stenotic lesions (OR = 1.35; p = 9.4 × 10^− 3^).

**Conclusions:**

Polygenic predisposition to CHD is strongly associated with atherosclerotic burden in individuals with type 2 diabetes and this effect is largely independent of traditional clinical risk factors. This suggests that genetic risk for CHD acts through atherosclerosis with little effect on most traditional risk factors, providing the opportunity to explore new biological pathways.

## Background

Coronary heart disease (CHD) is the leading cause of death in industrialized countries [1–3]. Type 2 diabetes increases both risk of CHD and CHD mortality by at least two-fold [4]. Consequently, individuals with type 2 diabetes undergo screening for other CHD risk factors and are often treated to reduce the burden of these risk factors [5, 6]. Current risk stratification strategies rely largely on traditional clinical risk factors, including blood pressure, serum lipids, poor glycemic control, obesity and smoking [7–9]. Among individuals with type 2 diabetes, these strategies fail to identify many individuals who will develop CHD, suggesting that risk of CHD in individuals with type 2 diabetes is driven through other risk factors.

One way to identify new risk factors for disease is to use modern human genetics approaches that determine which regions of the human genome are associated with risk of CHD [10]. The associations of single nucleotide polymorphisms (SNPs) with CHD can be used to construct a polygenic risk score (PRS) which aims to summarize information from common genetic variants across the genome to identify individuals at higher risk of CHD. Recently a PRS was constructed for prevalent CHD which identified 8% of people of European ancestry having a risk of CHD three-fold higher than the remaining 92% of the population [10]. This PRS was only weakly associated with known CHD risk factors, providing the opportunity to identify novel mechanisms influencing CHD risk. For example, having a PRS at the highest 5% of the population for CHD increased the odds of early onset myocardial infarction 3.7-fold, yet was only associated with a 6.0% higher LDL cholesterol, a 4.6% higher prevalence of hypertension, a 2.1% higher prevalence of type 2 diabetes and a 3.1% higher prevalence of smoking [11]. This suggests that much of the risk imparted by the PRS for CHD is acting through mechanisms independent of these risk factors. At present, these additional risk factors are largely unknown.

In order to better understand the mechanisms whereby this PRS is influencing risk of CHD specifically amongst individuals with type 2 diabetes, we tested the association of the CHD PRS with traditional clinical risk factors in participants with diabetes in the UK Biobank (N = 21,102). Furthermore, in the McGill Cardiac Complications in Diabetes (MCCD) cohort, which includes 352 individuals with type 2 diabetes who underwent coronary angiography, we evaluated whether the PRS for CHD was associated with multivessel stenotic disease and/or traditional CHD risk factors.

## Methods

### Cohorts and clinical risk factors

We obtained data from 437,192 participants of British ancestry (as indicated by the data field “ethnicity background”) from the UK Biobank [12]. Among these participants, 21,102 (4.8%) were reported to have been diagnosed with diabetes at the time of recruitment, as indicated by self-report of a physician-made diagnosis. Among these individuals with type 2 diabetes, prevalent CHD was determined by the criteria proposed by Khera et al. based on medical history of myocardial infarction or coronary revascularization [10]: 1898 (9.0%) had clinical records in at least one of the data fields with International Statistical Classification of Diseases and Related Health Problems (ICD)-9 codes of 410, 411.0, 412, or 429.79, or ICD-10 codes of I21, I23, I24.1, or I25.2, or procedure records in at least one of the data fields with Office of Population Censuses and Surveys (OPCS)-4 codes of K40.1–K40.4, K41.1–K41.4, K45.1–K45.5, K49.1–K49.2, K49.8–K49.9, K50.2, K75.1–K75.4 or K75.8–K75.9.

In order to test the association of the CHD PRS with CHD risk factors, we created binary variables for hypertension, lipids, poor glycemic control, obesity and smoking using clinically-relevant thresholds. Doing so enabled comparison of effect sizes of the CHD PRS across risk factors. Specifically, we first obtained systolic blood pressure (SBP), diastolic blood pressure (DBP), low density lipoprotein (LDL) and triglyceride (TG) levels from these same individuals. Following the 2019 Standards of Medical Care in Diabetes recommended by the American Diabetes Association (ADA). Then, we defined systolic hypertension as SBP ≥ 140 mmHg, diastolic hypertension as DBP ≥ 90 mmHg, high LDL as LDL ≥ 1.8 mmol/L and hypertriglyceridemia as TG ≥ 5.6 mmol/L [13]. Active usage of antihypertensive or lipid-lowering drugs may control blood pressure or blood lipid levels below these clinical cutoffs. Therefore, we further defined hypertension as having either systolic hypertension, diastolic hypertension, or taking antihypertensive drugs; and hyperlipidemia as having either high LDL, hypertriglyceridemia, or taking lipid-lowering drugs. Active medications were determined based on the data field “medication for cholesterol, blood pressure or diabetes”. Poor glycemic control was defined as having hemoglobin A1c (HbA1c) test level ≥ 8.0% (64 mmol/mol), corresponding to a less stringent HbA1c goal appropriate for patients with long-standing diabetes [13]. Obesity was defined as having a BMI ≥ 30 kg/m^2^. To generate a smoking history binary outcome, we used the data field “ever smoked”. We determined whether a sample had a family history of heart disease based on whether heart disease had been reported in any parent or sibling at the time of recruitment as indicated by data fields under the category “family history”. The UK Biobank has not quantified atherosclerotic burden.

The MCCD cohort was established between 2013 and 2015, by identifying patients undergoing coronary angiograms for clinical diagnosis or treatment who also had a diagnosis of type 2 diabetes by ADA criteria [14], which include fasting plasma glucose ≥ 7.0 mmol/L, or 2-h plasma glucose ≥ 11.1 mmol/L during a 75-g oral glucose tolerance test, or HbA1c ≥ 6.5% (48 mmol/mol), or random plasma glucose ≥ 11.1 mmol/L in patients with classic symptoms of hyperglycemia. Each individual underwent a clinically indicated coronary angiogram at one of two McGill University teaching hospitals: the Jewish General Hospital and the Royal Victoria Hospital. Blood pressure, blood lipid levels and anthropometric indices were measured at recruitment. Systolic hypertension, diastolic hypertension, high LDL, hypertriglyceridemia and obesity were defined using the same criteria described above for UK Biobank. Hypertension and hyperlipidemia were further determined in combination with self-reported active usage of antihypertensive drugs and lipid-lowering drugs. Self-reported current or previous smokers were regarded as having a smoking history. Participants who had at least one parent, sibling or child that had had a heart attack and/or congestive heart failure upon recruitment were regarded as having a family history of heart disease. All participants consented for participation in this study and the study was approved by the Research Ethics Board of McGill University.

### Definition of atherosclerotic burden in the MCCD cohort

As performed previously [15], we defined multivessel stenosis as having at least two lesions, each with ≥ 50% stenosis, influencing at least two of the four major coronary arteries (left main coronary artery, right coronary artery, left circumflex artery and left anterior descending artery). Each stenotic lesion was graded by a board-certified cardiologist who had additional training in angiography. Participants with a saphenous vein graft were considered to have multivessel stenosis. We also classified participants by the number of stenotic lesions (defined as lumen occlusion ≥ 50% in one of the four major coronary arteries): 0–1, 2, 3 and ≥ 4 lesions.

### Genotyping, genotype imputation and calculation of CHD PRS

All participants in the UK Biobank, were genome-wide genotyped using Affymetrix arrays [16] and their genotypes were imputed to the Haplotype Reference Consortium [17]. Genotyping details for UK Biobank have been described previously [16].

In the MCCD cohort, DNA was extracted and genome-wide genotyped using the Axiom Biobank array at the McGill University and Genome Quebec Innovation Centre. We excluded 14 samples with a genotyping call rate below 97.5%. We selected 541,272 markers that matched the human genome reference GRCh37 (hg19) from the 686,052 genotyped markers and used these markers for genotype imputation. We conducted pre-phasing and imputation using EAGLE2 [18] and PBWT [19] respectively, on the Sanger Imputation Service online computation platform (https://imputation.sanger.ac.uk. Accessed February 14, 2019). We chose the Haplotype Reference Consortium reference panel r1.1 [17] as reference since it has the largest set of haplotypes to enable imputation.

We next generated the CHD PRS, as developed by Khera et al. [10] using LDpred [20] to derive a CHD PRS for each sample in both cohorts. After imputation, we selected autosomal markers having an information quality value (imputation INFO score) > 0.3. 6012,299 (90.7%) in the diabetic UK Biobank cohort and 6262,956 (94.46%) in the MCCD cohort among the 6630,150 CHD PRS SNPs were genotyped or imputed and none of these SNPs contained missing genotypes. Since the CHD PRS does not contain any DNA polymorphisms with ambiguous strands (A/T or C/G), information from all imputed markers was utilized. We standardized the derived CHD PRS to have a mean of zero and a standard deviation of one in the two cohorts, respectively.

### Ethnicity estimation in the MCCD cohort

Owing to different patterns of linkage disequilibrium, allele frequency and genetic architecture, prediction in a different population other than the population on which the PRS was trained generally impairs accuracy [21]. While we retrieved a British-only diabetic population in the UK Biobank, the MCCD cohort contained samples of mixed ancestries. To define the ethnicity of each participant in the MCCD cohort, we first selected a representative subset of 162,811 SNPs from the genotyped and/or imputed autosomal markers. Selection of these genetically independent markers was performed by the linkage disequilibrium (LD)-based pruner implemented in PLINK version 1.9 [22] with argument–*indep*-*pairwise 50 5 0.5*. We next retrieved whole-genome genotyping information from 1668 participants of the 1000 Genomes Project with defined ancestry: 661 Africans, 504 East Asians and 503 Europeans [23]. The same 162,811 SNPs were used in these individuals. We combined the 352 samples in our study with the 1668 reference samples and performed principal component analysis using R package SNP Relate version 3.8 [24]. We assigned putative ancestry (European/Non-European) to each sample based on overlap with the corresponding population. Our primary analyses included people of European ancestry since this was the largest population cluster. All analyses were then repeated including individuals of non-European ancestry.

### Association study of CHD PRS with CHD and traditional clinical risk factors

In the UK Biobank cohort, we first tested the association between CHD PRS and CHD amongst individuals with type 2 diabetes using a logistic regression model adjusting for fixed effects of age and sex. In both the UK Biobank and the MCCD cohorts, we then tested for associations between CHD PRS and traditional clinical risk factors for CHD. We adopted logistic regression models to test for associations between CHD PRS and hypertension, systolic hypertension, diastolic hypertension, hyperlipidemia, high LDL, hypertriglyceridemia, poor glycemic control, obesity, smoking and family history of heart disease respectively. For continuous traits, we also derived the standardized beta coefficients using linear regression models. Tests performed on the diabetic UK Biobank cohort were adjusted for sex and age; in the MCCD cohort, analyses were adjusted for sex, age and hospital of recruitment; analyses separately by sex were also performed.

### Analysis of CHD PRS and atherosclerosis in the MCCD cohort

We performed logistic regression using multivessel stenosis as the outcome, as well as ordinal logistic regression using graded atherosclerosis severity based on the number of atherosclerotic lesions as the outcome (described above). Both analyses were performed among samples of putative European ancestry and repeated using all samples using sex, age and hospital of recruitment as covariates. To assess potential hospital-based effects we repeated analyses separately for each hospital. To better address the effect of CHD PRS on samples recruited at different hospitals, we meta-analyzed the coefficients of the CHD PRS in the aforementioned logistic and ordinal logistic regression models using a linear mixed-effect model implemented in the *rma.uni* function of the R package metafor version 2.0-0 [25].

## Results

### Clinical characteristics

Among 21,102 participants of British ancestry diagnosed with type 2 diabetes upon recruitment to UK Biobank, the median age was 62 and the majority were men (61.7%). The prevalence of CHD at recruitment was 9.0%. Apart from hypertriglyceridemia (2.0%), traditional clinical risk factors for CHD were common (Table 1).

**Table 1:** Clinical characteristics in the population of British ancestry in the UK Biobank with type 2 diabetes

	Participants (n = 21,102)	Participants with information (%)
CHD (%)	1898 (9.0)	21,102 (100)
Mean CHD PRS^a^ (SD)	16.49 (0.08)	21,102 (100)
Men (%)	13,010 (61.7)	21,102 (100)
Median age (IQR)	62 (56–66)	21,102 (100)
Hypertension (%)	14,899 (70.6)	21,102 (100)
Systolic hypertension (%)	10,837 (54.6)	19,866 (94.1)
Diastolic hypertension (%)	3551 (17.9)	19,866 (94.1)
Antihypertensive drug usage (%)	8697 (41.2)	21,102 (100)
Hyperlipidemia (%)	19,964 (94.6)	21,102 (100)
High LDL (%)	18,280 (91.2)	20,035 (94.9)
Hypertriglyceridemia (%)	418 (2.0)	20,055 (95.0)
Lipid-lowering drug usage (%)	10,077 (47.8)	21,102 (100)
Poor glycemic control (%)	3406 (16.9)	20,171 (95.6)
Median BMI (IQR)	30.80 (27.50–34.80)	20,347 (96.4)
Smoking history (%)	14,039 (66.9)	20,971 (99.4)
Family history of heart disease (%)	10,560 (50.0)	21,102 (100)

^a^ Non-standardized PRS is reported in this table to allow comparison with other cohorts. All subsequent analyses adopted standardized PRS

In the MCCD cohort, there were 367 patients initially recruited who met ADA diagnostic criterion for type 2 diabetes. Fourteen samples were removed after genotyping quality control and one sample was removed due to lack of clinical phenotypes. The resulting sample size was thus, 352 (Table 2). The median age was 71 and 76.4% were men. Multivessel stenosis was identified in 67.1% of the cohort. Eighty-eight percent (88.1%) of the samples were found to be of European ancestry (Additional file 1: Figure S1).

**Table 2:** Clinical characteristics in the MCCD cohort

	Patients (n = 352)	Patients with information (%)
Multivessel stenosis (%)	236 (67.1)	352 (100)
Median number of significant lesions (IQR)	2 (1–4)	352 (100)
Severity of atherosclerosis		352 (100)
0–1 (%)	116 (33.0)	
2 (%)	68 (19.3)	
3 (%)	61 (17.3)	
≥ 4 (%)	107 (30.4)	
Mean PRS^a^ (SD)	17.18 (0.10)	352 (100)
Men (%)	269 (76.4)	352 (100)
Median age (IQR)	71 (66–78)	352 (100)
European (%)	310 (88.1)	352 (100)
Hypertension (%)	328 (93.2)	352 (100)
Systolic hypertension (%)	149 (42.7)	349 (99.1)
Diastolic hypertension (%)	16 (4.6)	349 (99.1)
Antihypertensive drug usage (%)	311 (88.4)	352 (100)
Hyperlipidemia (%)	333 (94.9)	351 (99.7)
High LDL (%)	146 (42.8)	341 (96.9)
Hypertriglyceridemia (%)	2 (0.5)	347 (98.6)
Lipid-lowering drug usage (%)	296 (84.1)	352 (100)
Poor glycemic control (%)	48 (14.0)	343 (97.4)
Median BMI (IQR)	29.82 (26.15–33.90)	346 (98.3)
Ex- or current smokers^b^ (%)	197 (56.0)	352 (100)
Median type 2 diabetes duration^b^ (IQR)	15 (8–22)	189 (53.7)
Median age of type 2 diabetes diagnosis^b^ (IQR)	58 (47–64)	189 (53.7)
Family history of heart disease^b^ (%)	209 (73.9)	283 (83.4)

^a^Original PRS is reported in this table. Downstream analyses adopted standardized PRS

^b^Based on self-reported medical histories

With the exceptions of diastolic hypertension (0.5%) and hypertriglyceridemia (4.6%), clinical risk factors for CHD were common in the MCCD cohort (Table 2). Participants had been diagnosed with type 2 diabetes for a median of 15 years. Fourteen percent of the samples had poor glycemic control. Demographic characteristics were essentially consistent between patients recruited at the two different hospitals. Despite a higher proportion of patients at the Jewish General Hospital with systolic hypertension and high LDL, the two hospitals had equally prominent high prevalence of hypertension (93.1% at the Jewish General Hospital; 93.5% at the Royal Victoria Hospital) and hyperlipidemia (94.9% at the Jewish General Hospital; 94.8% at the Royal Victoria Hospital) (Additional file 1: Tables S1, S2).

### CHD PRS is strongly associated with CHD risk amongst participants with type 2 diabetes in UK Biobank and MCCD

**Fig. 1:**
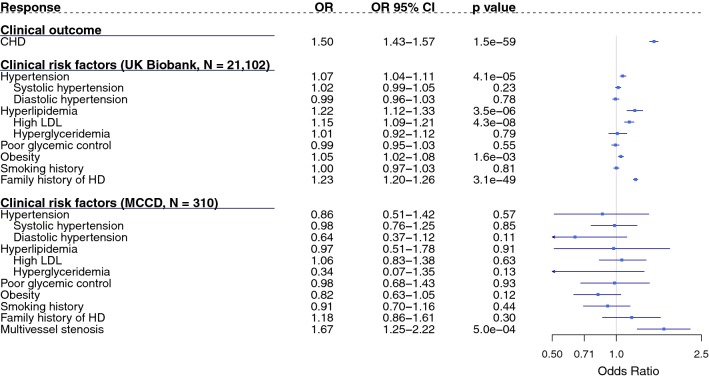
Associations of CHD PRS with CHD and clinical risk factors among individuals of European ancestry with type 2 diabetes. Associations were tested using logistic regression model adjusted for age and sex. Results presented are based on diabetic participants of British ancestry in the UK Biobank (N = 21,102) or participants of European ancestry in the MCCD cohort (N = 310). *HD* heart disease, *OR* odds ratio, *CI* confidence interval. ORs are presented on a logarithmic scale as squares with corresponding CIs indicated by solid lines. Arrows indicate CIs beyond the illustrated range

Amongst individuals with type 2 diabetes in the UK Biobank cohort, a standard deviation increase in CHD PRS was found to be associated with a 1.50-fold [95% Confidence Interval (CI) 1.43–1.57; p = 1.5 × 10^− 59^] increased odds of CHD (Fig. 1). The magnitude of this association was consistent in both women and men, with a 1.45-fold and a 1.51-fold increased odds per standard deviation increase in CHD PRS, respectively (Additional file 1: Figure S2). Adjusting for traditional clinical risk factors slightly altered the odds of CHD associated with the CHD PRS [Odds Ratio (OR) = 1.46; 95% CI 1.38–1.54; p = 2.3 × 10^− 43^, Additional file 1: Table S3].

### An increase in CHD PRS was not strongly associated with common risk factors in either UK Biobank or MCCD

In the diabetic UK Biobank cohort, a high CHD PRS was not strongly correlated with hypertension, hypertriglyceridemia, poor glycemic control, obesity and smoking history and the 95% CI’s overlapped or were very close to the null (Fig. 1). However, a standard deviation increase in the CHD PRS was associated with a 1.22-fold (95% CI 1.12–1.33; p = 3.5 × 10^− 6^) increased odds of hyperlipidemia and a 1.15-fold (95% CI 1.09–1.21; p = 4.3 × 10^−8^) increased odds of presence of high LDL (Fig. 1). A high CHD PRS was also associated with an increased odds of having a family history of heart disease (OR per standard deviation increase = 1.23; 95% CI 1.20–1.26; p = 3.1 × 10^−49^; Fig. 1). Results were very similar in both women and men in this cohort (Additional file 1: Figure S2).

Among samples of European ancestry in the MCCD cohort (88.1% of the cohort), estimates of the same associations were less precise with wider confidence intervals. Nevertheless, either for the whole cohort or for the European subset, the association of the CHD PRS with all of the traditional risk factors included the null value within their CIs (Fig. 1 and Additional file 1: Table S4).

In both cohorts, we also found weak associations between the CHD PRS and clinical risk factors when they were treated as continuous risk factors by using standardized beta coefficients in linear models (Additional file 1: Table S5). For instance, in the diabetic UK Biobank cohort, LDL showed the strongest association with the CHD PRS, yet one standard deviation increase in the CHD PRS was only associated with 3.4% standard deviation increase in LDL level (Additional file 1: Table S5).

### Increase in CHD PRS was associated with increased atherosclerotic burden in patients with type 2 diabetes in MCCD

Among samples of European ancestry in the MCCD cohort, a higher CHD PRS was strongly associated with increased risk of multivessel stenosis, where a standard deviation increase in the CHD PRS increased the odds of multivessel stenosis 1.65-fold (95% CI 1.25–2.20, p = 4.9 × 10^−4^) (Table 3). This strong association was observed in both 65 women and 245 men, with an increased odds of multivessel stenosis of 2.37-fold (95% CI 0.99–5.64, p = 5.1 × 10^−2^) and 1.49-fold (95% CI 1.06–2.09, p = 2.2 × 10^−2^), respectively. This association was attenuated upon introduction of individuals of non-European ancestry, but the confidence intervals still excluded the null (OR per standard deviation increase in CHD PRS = 1.46; 95% CI 1.14-1.87; p = 2.8 × 10^−3^; Additional file 1: Table S6). Based on severity grading of atherosclerosis, 116, 68, 61 and 107 patients had 0–1, 2, 3, and ≥ 4 significant atherosclerotic lesions, respectively (Table 1). A higher CHD PRS was also associated with an increased severity of atherosclerosis in both the European-ancestry population (OR per standard deviation increase in CHD PRS = 1.35; 95% CI 1.08–1.69; p = 9.4 × 10^−3^; Table 3) and the mixed ancestry population (OR per standard deviation increase in CHD PRS = 1.29; 95% CI 1.05–1.57; p = 1.4 × 10^−2^; Additional file 1: Table S6).

**Table 3:** Risk factors for atherosclerosis among individuals of European ancestry in the MCCD cohort

	OR^a^ for atherosclerosis	OR 95% C.I.	p value
Multivessel stenosis			
CHD PRS (per SD increase)	1.65	1.25–2.20	4.9e–04
Men	3.32	1.84-6.08	8.0e–05
Age (per year increase)	1.03	1.00–1.06	3.8e–02
Royal Victoria Hospital^b^	0.40	0.22–0.73	2.7e–03
Graded atherosclerosis severity			
CHD PRS (per SD increase)	1.35	1.08–1.69	9.4e–03
Men	3.22	1.91–5.50	1.3e–05
Age (per year increase)	1.02	1.00–1.04	6.0e–02
Royal Victoria Hospital	0.39	0.23–0.64	2.2e–04

^a^Odds ratio estimated by multivariate logistic regression, based on 310 individuals with a European ancestry

^b^Being recruited at the Royal Victoria Hospital

## Discussion

In this study, we demonstrated that a CHD PRS was associated with CHD risk in 21,102 individuals with prevalent diabetes in the UK biobank conferring a 50% increase in the odds of CHD per standard deviation increase in the PRS. We also show that the PRS did not associate with most traditional atherosclerotic risk factors, although a modest association was identified with hyperlipidemia and elevated LDL. Conversely, the PRS for CHD was strongly associated with severity of atherosclerosis in individuals with type 2 diabetes, in a cohort of diabetic patients where angiograms and genotyping had both been performed. These findings suggest that a major mechanism whereby genetic risk influences CHD risk in individuals with diabetes is through increased atherosclerotic burden.

Recent large-scale genome-wide association studies have demonstrated that CHD and type 2 diabetes have a shared genetic background [26]. For example, concomitant presence of CETP B1, NOS3 T and ANGPTL8 T alleles augments the risk of both CHD and type 2 diabetes [27]. It has also been shown that genetic predisposition to type 2 diabetes is significantly associated with greater severity of coronary atheromatous burden in patients with acute coronary syndrome, independently of traditional risk factors [28].

Our results indicate that, mechanistically, the PRS likely confers increased cardiovascular risk in individuals with type 2 diabetes by accelerating the development of atherosclerosis. This result, therefore, plausibly represents an important contributor to the advanced burden of atherosclerotic disease often observed in individuals with type 2 diabetes. These results are noteworthy as they suggest that despite the metabolic consequences of diabetes which are known to accelerate vascular disease via traditional mechanisms (i.e. elevated atherogenic lipoproteins, dysglycemia, obesity, etc.), a genetic predisposition remains an independent and potentially clinically relevant factor that appears to act in large part through atherosclerotic burden. This association is similar to what has been observed in a general population [29].

Our findings may also have implications for using PRS to guide understanding of CHD pathogenesis in the presence of long-term diabetes or other co-occurring systemic diseases. Polygenic risk scores present an opportunity to understand the mechanisms that underlie disease causation via other mechanisms not related to traditional risk factors. Such approaches may be particularly helpful in understanding the causes of CHD in type 2 diabetes since the distinct biological mechanisms are unclear and such patients have more accelerated and progressive disease, providing an opportunity to better understand the mechanisms underlying this predisposition. We identified that hyperlipidemia had a modest association with the CHD PRS. This is not entirely surprising given the CHD PRS includes SNPs in pathways relevant to hyperlipidemia, such as two influential SNPs (rs186696265 and rs10455872) residing near the *LPA* gene [30–32], which is known to increase levels of lipoprotein (a), a well-established atherogenic lipoprotein. However, the effect of CHD PRS on CHD risk does not appear to be predominantly mediated by hyperlipidemia since a standard increase in CHD PRS adjusted for hyperlipidemia still incurs a 1.49-fold increased odds of CHD, which implies that hyperlipidemia is only one of many factors influencing risk of CHD. Whether the CHD PRS predisposes to other mechanisms that interact synergistically with the metabolic derangements in diabetes to either promote atherogenic particle retention in the intima, via oxidation, glycation or other mechanisms, warrants further study.

### A CHD PRS could potentially help to identify individuals with type 2 diabetes at an increased risk of CHD

Building on the accumulating studies of the genetic mechanisms underlying CHD, efforts have been made to incorporate knowledge of genetic risk into clinical decision-making [33]. For example, a prospective study including four statin trials showed that CHD PRSs could identify individuals who would benefit the most from statin therapy [34]; Another randomized controlled trial showed that genetically-informed decision-making could lead to reduced LDL levels [35]. Our study may also have direct clinical implications for the prevention of CHD in individuals with type 2 diabetes. Due to medication usage, chronic metabolic disorders, and other complications, many established associations between clinical risk factors and pathogenetic mechanisms may be attenuated. For instance, in our angiographic cohort where the duration of type 2 diabetes is generally long and usage of lipid-lowering drugs is prevalent, high LDL is not associated with multivessel stenosis (OR = 0.97; 95% CI 0.59–1.61; p = 0.91). In contrast, the genetic mechanisms likely remain persistent and have been shown to be promising in identifying individuals with type 2 diabetes at a high CHD risk [36, 37]. In this study, we show that a CHD PRS, capturing more genetic risk than previous genetic risk scores, maintains its association. Therefore, although several issues remain prior to translation to clinical use including setting reasonable clinical cutoffs for the normally distributed PRS and incorporating other clinical or lifestyle risk factors into an integrative risk assessment system, we posit that the CHD PRS is promising to improve CHD risk stratification and prevention amongst patients with long-standing type 2 diabetes as it provides sufficient predictive performance [10].

## Limitations and future directions

Our study has important limitations. The definition of type 2 diabetes in the UK Biobank was based on self-reported physician-made diagnosis instead of objective glucose or HbA1c levels, which may fail to include all patients with type 2 diabetes. However, this cohort still has a prominently large sample size with high reliability. It is also worth noting that that a genome-wide PRS developed in a European population can accurately predict CHD in a separate population of French Canadians [38], the effect of the CHD PRS in individuals of non-European ancestry has not been well-studied [33]. Further studies of different ancestries will be required to understand if the effects of the CHD PRS on atherosclerotic burden are seen in other populations. Nevertheless, the effect size of CHD PRS is only slightly smaller in the mixed ancestry population, when compared to individuals of only European ancestry (Additional file 1: Table S6).

There was a between-hospital difference in the MCCD cohort. Patients recruited at the Royal Victoria Hospital were apparently less likely to have developed multivessel stenosis (Additional file 1: Tables S1, S2), even though the estimated ORs associated with one standard deviation increase in CHD PRS for both multivessel stenosis and the count of atherosclerotic lesions were similar between hospitals (Additional file 1: Table S7). Nevertheless, our results are consistent when hospital-specific ORs were calculated and combined by a meta-analysis (Additional file 1: Figure S3).

While the age of CHD onset is usually ascertainable based on interviews or medical records, the onset of type 2 diabetes can be gradual and free of symptoms making timing of disease onset difficult. Therefore, we did not opt to quantify an age-dependent risk in the UK Biobank. On the other hand, duration of type 2 diabetes varies among the MCCD cohort and both developing type 2 diabetes at a younger age and having a longer duration of type 2 diabetes seem to increase atherosclerosis risk (Additional file 1: Table S8). These results are directionally consistent with previously-reported CHD-type 2 diabetes progression associations [39–41]. The magnitude of the impact of the PRS on risk of atherosclerosis may vary with type 2 diabetes duration but this will need to be investigated in future studies, given our lack of power to address this question.

Sex/gender differences have been reported in patients with atherosclerotic cardiovascular diseases [42]. Importantly, women with diabetes have been reported to be at greater risk for CHD and more likely experience adverse outcomes [43]. In this study, we observed that the associations between polygenic risk for CHD and risk factors were not modified by sex differences, since all of the 95% CIs substantially overlapped. Since the PRS was derived from alleles on the autosomes, this is of course expected. We posit that observed sex differences in CHD pathogenesis, progression and prognosis may therefore be more attributable to various sex chromosome, epigenetic regulatory and environmental mechanisms yet to be illustrated. However, we recognize that our estimates of sex-specific effect sizes are imprecise due to the smaller sample sizes, particularly in the MCCD cohort. We believe studies based on larger cohorts in the future will help validate and extend our findings while providing more precise estimates of effect sizes.

## Conclusions

In conclusion, a PRS for CHD is strongly associated with odds of CHD amongst individuals with type 2 diabetes with no discernible association for most CHD risk factors, except hyperlipidemia. The CHD PRS is strongly associated with atherosclerotic burden indicating that the PRS predisposes to accelerated atherosclerosis in individuals with type 2 diabetes via apparently novel pathways.

## Supplementary information

**Additional file 1: Table S1.** Characteristics of patients recruited at the Jewish General Hospital. **Table S2.** Characteristics of patients recruited at the Royal Victoria Hospital. **Table S3**. Effect of CHD PRS on CHD adjusted for traditional clinical risk factors in the diabetic UK Biobank cohort. **Table S4.** Associations of CHD PRS with clinical risk factors among all 352 individuals in MCCD. **Table S5.** Association between CHD PRS and continuous clinical risk factors in the diabetic UK Biobank cohort and the MCCD cohort. **Table S6.** Effect of non-European ancestry in MCCD. **Table S7.** Effect of CHD PRS by hospital among European descendants in the MCCD cohort. **Table S8.** Effects of type 2 diabetes duration and age of onset on atherosclerotic burden among European descendants in the MCCD cohort. **Figure S1.** Ethnicity decomposition of local population. 162,811 representative LDpruned SNPs were used to examine similarity of each individual with the Eastern Asian (EAS), African (AFR) and European (EUR) populations from the 1000 Genomes Project (G1000 cohort). Samples residing in the shaded area were regarded as European descendants. Samples residing in the middle of two super populations may have an unlisted ancestry or a mixed ancestry, and were thus not regarded as European descendants Samples recruited at the two different hospitals were shaped differently. AFR: the African super population; EAS: The East Asian super population; EUR: The European super population; MCCD: The McGill Cardiac Complications in Diabetes cohort. **Figure S2.** Sex-specific associations of CHD PRS with CHD and clinical risk factors among individuals with type 2 diabetes. (a) Female-specific associations based on 8092 women in the diabetic UK Biobank cohort and 65 women of a European ancestry in the MCCD cohort. (b) Male-specific associations based on 13,010 men in the diabetic UK Biobank cohort and 245 men of a European ancestry in the MCCD cohort. All results were based on logistic regression models as presented in Fig. 1 without adjusting for sex. **Figure S3.** Meta-analyses of ORs based on (a) logistic regression models using multivessel stenosis as the outcome and (b) ordinal logistic regression models using the number of atherosclerotic lesions as the outcome. Regressions were performed separately by hospital on samples with a 0putative European ancestry in the MCCD cohort. Estimated ORs are represented by squares where the area is proportional to the corresponding sample size at each hospital. Metaanalytic ORs are represented by diamonds. For all ORs, the corresponding CIs are indicated inside brackets. JGH: The Jewish General Hospital; RVH: The Royal Victoria Hospital.

## Data Availability

The data that support the findings of this study are available from the corresponding author upon reasonable request.

## Abbreviations

ADA: American diabetes association
BMI: body mass index
CHD: coronary heart disease
CI: confidence interval
DBP: diastolic blood pressure
HbA1c: hemoglobin A1c
ICD: International Statistical Classification of Diseases and Related Health Problems
LD: linkage disequilibrium
LDL: low density lipoprotein
MCCD: McGill Cardiac Complications in Diabetes cohort
OPCS: Office of Population Censuses and Surveys
OR: odds ratio
PRS: polygenic risk score
SBP: systolic blood pressure
SNP: single nucleotide polymorphism
TG: triglyceride
