# Supplementary material for: Polygenic risk for coronary heart disease acts through atherosclerosis in type 2 diabetes
Source: Cardiovasc Diabetol. 2020 Jan 30;19:12. doi: 10.1186/s12933-020-0988-9 (PMC6993460; doi:10.1186/s12933-020-0988-9)
Supplement: Supplementary file 1 — Additional file 1: Table S1. Characteristics of patients recruited at the Jewish General Hospital. Table S2. Characteristics of patients recruited at the Royal Victoria Hospital. Table S3. Effect of CHD PRS on CHD adjusted for traditional clinical risk factors in the diabetic UK Biobank cohort. Table S4. Associations of CHD PRS with clinical risk factors among all 352 individuals in MCCD. Table S5. Association between CHD PRS and continuous clinical risk factors in the diabetic UK Biobank cohort and the MCCD cohort. Table S6. Effect of non-European ancestry in MCCD. Table S7. Effect of CHD PRS by hospital among European descendants in the MCCD cohort. Table S8. Effects of type 2 diabetes duration and age of onset on atherosclerotic burden among European descendants in the MCCD cohort. Figure S1. Ethnicity decomposition of local population. 162,811 representative LDpruned SNPs were used to examine similarity of each individual with the Eastern Asian (EAS), African (AFR) and European (EUR) populations from the 1000 Genomes Project (G1000 cohort). Samples residing in the shaded area were regarded as European descendants. Samples residing in the middle of two super populations may have an unlisted ancestry or a mixed ancestry, and were thus not regarded as European descendants Samples recruited at the two different hospitals were shaped differently. AFR: the African super population; EAS: The East Asian super population; EUR: The European super population; MCCD: The McGill Cardiac Complications in Diabetes cohort. Figure S2. Sex-specific associations of CHD PRS with CHD and clinical risk factors among individuals with type 2 diabetes. (a) Female-specific associations based on 8092 women in the diabetic UK Biobank cohort and 65 women of a European ancestry in the MCCD cohort. (b) Male-specific associations based on 13,010 men in the diabetic UK Biobank cohort and 245 men of a European ancestry in the MCCD cohort. All results were based on logistic regression models as pr [file 12933_2020_988_MOESM1_ESM.docx]

**ADDITIONAL FILE**

**Polygenic risk for coronary heart disease acts through atherosclerosis in type 2 diabetes**

Tianyuan Lu, Vincenzo Forgetta, Oriana HY Yu, Lauren Mokry, Madeline Gregory, George Thanassoulis, Celia MT Greenwood and J Brent Richards

**Supplemental Table S1. Characteristics of patients recruited at the Jewish General Hospital**

|  | Patients (n=275) | Patients with information (%) |
| --- | --- | --- |
| Multivessel stenosis (%) | 194 (70.6) | 275 (100) |
| Median number of significant lesions (IQR) | 3 (1-4) | 275 (100) |
| Severity of atherosclerosis |  | 77 (100) |
| 0-1 (%) | 81 (29.5) |  |
| 2 (%) | 53 (19.3) |  |
| 3 (%) | 46 (16.7) |  |
| ≥4 (%) | 95 (34.5) |  |
| Mean PRS (SD) | 17.18 (0.10) | 275 (100) |
| Men (%) | 203 (73.8) | 275 (100) |
| Median age (IQR) | 71 (66-79) | 275 (100) |
| European (%) | 242 (88.0) | 275 (100) |
| Hypertension (%) | 256 (93.1) | 275 (100) |
| Systolic hypertension (%) | 128 (46.9) | 273 (99.3) |
| Diastolic hypertension (%) | 15 (5.6) | 273 (99.3) |
| Antihypertensive drug usage (%) | 241 (87.6) | 275 (100) |
| Hyperlipidemia (%) | 260 (94.9) | 274 (99.6) |
| High LDL (%) | 125 (47.0) | 266 (96.7) |
| Hypertriglyceridemia (%) | 2 (0.7) | 270 (98.2) |
| Lipid-lowering drug usage (%) | 227 (82.5) | 275 (100) |
| Poor glycemic control (%) | 35 (13.2) | 266 (96.7) |
| Median BMI (IQR) | 30.00 (26.30-34.28) | 270 (98.2) |
| Ex- or current smokers (%) | 152 (55.3) | 275 (100) |
| Median type 2 diabetes duration (IQR) | 14 (7-22) | 137 (49.8) |
| Median age of type 2 diabetes diagnosis (IQR) | 59 (48-65) | 137 (49.8) |
| Family history of heart disease (%) | 163 (73.1) | 223 (81.1) |

**Supplemental Table S2. Characteristics of patients recruited at the Royal Victoria Hospital**

|  | Patients (n=77) | Patients with information (%) |
| --- | --- | --- |
| Multivessel stenosis (%) | 42 (54.6) | 77 (100) |
| Median number of significant lesions (IQR) | 2 (0-3) | 77 (100) |
| Severity of atherosclerosis |  | 77 (100) |
| 0-1 (%) | 35 (45.5) |  |
| 2 (%) | 15 (19.5) |  |
| 3 (%) | 15 (19.5) |  |
| ≥4 (%) | 12 (15.6) |  |
| Mean PRS (SD) | 17.21 (0.09) | 77 (100) |
| Men (%) | 66 (85.7) | 77 (100) |
| Median age (IQR) | 73 (64-77) | 77 (100) |
| European (%) | 68 (88.3) | 77 (100) |
| Hypertension (%) | 72 (93.5) | 77 (100) |
| Systolic hypertension (%) | 21 (27.6) | 76 (98.7) |
| Diastolic hypertension (%) | 1 (1.3) | 76 (98.7) |
| Antihypertensive drug usage (%) | 70 (90.9) | 77 (100) |
| Hyperlipidemia (%) | 73 (94.8) | 77 (100) |
| High LDL (%) | 21 (28.0) | 75 (97.4) |
| Hypertriglyceridemia (%) | 0 (0) | 77 (100) |
| Lipid-lowering drug usage (%) | 69 (89.6) | 77 (100) |
| Poor glycemic control (%) | 13 (16.9) | 77 (100) |
| Median BMI (IQR) | 29.60 (26.08-31.82) | 76 (98.7) |
| Ex- or current smokers (%) | 45 (58.4) | 77 (100) |
| Median type 2 diabetes duration (IQR) | 15 (10-21) | 52 (67.5) |
| Median age of type 2 diabetes diagnosis (IQR) | 55 (44-61) | 52 (67.5) |
| Family history of heart disease (%) | 46 (76.7) | 60 (77.9) |

**Supplemental Table S3. Effect of CHD PRS on CHD adjusted for traditional clinical risk factors in the diabetic UK Biobank cohort**

|  | OR^*^ | OR 95% CI | p value |
| --- | --- | --- | --- |
| CHD PRS | 1.46 | 1.38-1.54 | 2.3e-43 |
| Men | 2.51 | 2.18-2.90 | 7.1e-37 |
| Age | 1.06 | 1.05-1.07 | 1.2e-33 |
| Hypertension | 1.25 | 1.08-1.46 | 3.6e-03 |
| Hyperlipidemia | 1.16 | 0.78-1.83 | 0.48 |
| Poor glycemic control | 1.55 | 1.36-1.77 | 7.4e-11 |
| Obesity | 1.31 | 1.17-1.46 | 1.4e-06 |
| Smoking history | 1.51 | 1.39-1.64 | 1.5e-22 |
| Family history of HD | 1.78 | 1.60-1.99 | 3.0e-25 |

^*^: Odds ratio of prevalent CHD associated with each risk factor was estimated by a multivariate logistic regression model including fixed effects of CHD PRS, sex, age and all traditional clinical risk factors listed

**Supplemental Table S4. Associations of CHD PRS with clinical risk factors among all 352 individuals in MCCD.**

|  | OR^*^ | OR 95% CI | p value |
| --- | --- | --- | --- |
| Hypertension | 1.02 | 0.66-1.57 | 0.92 |
| Systolic hypertension | 1.05 | 0.84-1.30 | 0.68 |
| Diastolic hypertension | 0.78 | 0.48-1.30 | 0.34 |
| Hyperlipidemia | 1.32 | 0.82-2.13 | 0.25 |
| High LDL | 1.03 | 0.82-1.29 | 0.80 |
| Hyperglyceridemia | 0.45 | 0.09-1.62 | 0.23 |
| Poor glycemic control | 0.98 | 0.72-1.34 | 0.90 |
| Obesity | 0.86 | 0.69-1.06 | 0.17 |
| Smoking history | 1.07 | 0.86-1.33 | 0.55 |
| Family history of HD | 1.24 | 0.95-1.62 | 0.12 |

^*^: Odds ratio of prevalent CHD associated with each risk factor was estimated by a multivariate logistic regression model including fixed effects of CHD PRS, sex, age, and ancestry

**Supplemental Table S5. Association between CHD PRS and continuous clinical risk factors in the diabetic UK Biobank cohort and the MCCD cohort**

|  | β^*^ (Standard error) | Adjusted R^2^ | p value |
| --- | --- | --- | --- |
|  |  |  |  |
| Clinical risk factors (UK Biobank) |  |  |  |
| Systolic blood pressure | 1.3e-02 (6.9e-03) | 5.3e-02 | 6.1e-02 |
| Diastolic blood pressure | -2.1e-02 (7.0e-03) | 2.5e-02 | 2.3e-03 |
| LDL | 3.4e-02 (6.9e-03) | 4.0e-02 | 8.2e-07 |
| Triglycerides | -1.1e-05 (7.0e-03) | 4.7e-03 | 1.00 |
| HbA1c | 3.2e-04 (7.0e-03) | 4.5e-03 | 0.96 |
| BMI | 2.2e-02 (7.0e-03) | 1.4e-02 | 1.4e-03 |
|  |  |  |  |
| Clinical risk factors (MCCD^†^) |  |  |  |
| Systolic blood pressure | 7.6e-02 (6.1e-02) | 4.3e-02 | 0.21 |
| Diastolic blood pressure | -5.5e-02 (5.9e-02) | 9.0e-02 | 0.35 |
| LDL | 2.8e-02 (6.1e-02) | 7.0e-02 | 0.65 |
| Triglycerides | -7.6e-02 (6.2e-02) | 1.5e-02 | 0.22 |
| HbA1c | -3.4e-02 (6.2e-02) | 1.7e-02 | 0.57 |
| BMI | -0.12 (6.0e-02) | 8.0e-02 | 4.2e-02 |

^*^: Standardized beta coefficients

^†^: 310 European descendants only

**Supplemental Table S6. Effect of non-European ancestry in MCCD**

|  | OR^*^ | OR 95% CI | p value |
| --- | --- | --- | --- |
|  |  |  |  |
| Multivessel stenosis |  |  |  |
| PRS (per SD increase) | 1.46 | 1.14-1.87 | 2.8e-03 |
| Men | 3.08 | 1.79-5.32 | 5.0e-05 |
| Age (per year increase) | 1.02 | 0.99-1.04 | 0.13 |
| Royal Victoria Hospital | 0.36 | 0.21-0.63 | 3.4e-04 |
| Non-European ancestry | 1.49 | 0.71-3.25 | 0.30 |
|  |  |  |  |
| Graded atherosclerosis severity |  |  |  |
| PRS (per SD increase) | 1.29 | 1.05-1.57 | 1.4e-02 |
| Men | 2.96 | 1.84-4.80 | 8.9e-06 |
| Age (per year increase) | 1.02 | 1.00-1.04 | 0.13 |
| Royal Victoria Hospital | 0.37 | 0.23-0.60 | 5.5e-05 |
| Non-European ancestry | 1.44 | 0.75-2.78 | 1.4e-02 |

^*^: Odds ratio estimated by multivariate logistic regression

**Supplemental Table S7. Effect of CHD PRS by hospital among European descendants in the MCCD cohort**

|  |  | OR^*^ | OR 95% CI | p value |
| --- | --- | --- | --- | --- |
|  |  |  |  |  |
| Jewish General Hospital |  |  |  |  |
|  | Multivessel stenosis |  |  |  |
|  | PRS (per SD increase) | 1.57 | 1.15-2.17 | 5.5e-03 |
|  | Men | 3.57 | 1.89-6.82 | 9.3e-05 |
|  | Age (per year increase) | 1.02 | 0.99-1.05 | 0.14 |
|  |  |  |  |  |
|  | Graded atherosclerosis severity |  |  |  |
|  | PRS (per SD increase) | 1.25 | 0.98-1.61 | 7.6e-02 |
|  | Men | 3.35 | 1.92-5.96 | 2.8e-05 |
|  | Age (per year increase) | 1.02 | 1.00-1.04 | 0.12 |
|  |  |  |  |  |
| Royal Victoria Hospital |  |  |  |  |
|  | Multivessel stenosis |  |  |  |
|  | PRS (per SD increase) | 2.08 | 1.11-4.19 | 2.8e-02 |
|  | Men | 2.60 | 0.48-15.37 | 0.27 |
|  | Age (per year increase) | 1.05 | 0.99-1.13 | 0.12 |
|  |  |  |  |  |
|  | Graded atherosclerosis severity |  |  |  |
|  | PRS (per SD increase) | 1.90 | 1.09-3.43 | 2.7e-02 |
|  | Men | 2.84 | 0.66-14.09 | 0.17 |
|  | Age (per year increase) | 1.04 | 0.98-1.10 | 0.23 |

^*^: Odds ratio estimated by multivariate logistic regression

**Supplemental Table S8. Effects of type 2 diabetes duration and age of onset on atherosclerotic burden among European descendants in the MCCD cohort**

|  | OR | OR 95% CI | p value |
| --- | --- | --- | --- |
|  |  |  |  |
| Including effect of type 2 diabetes duration^*^ |  |  |  |
| PRS (per SD increase) | 1.77 | 1.20-2.69 | 5.0e-03 |
| Men | 3.02 | 1.28-7.28 | 1.2e-02 |
| Age (per year increase) | 1.05 | 1.01-1.10 | 2.3e-02 |
| Royal Victoria Hospital | 0.34 | 0.15-0.77 | 1.1e-02 |
| 11-20 years | 1.33 | 0.60-2.98 | 0.48 |
| >20 years | 1.65 | 0.67-4.26 | 0.28 |
|  |  |  |  |
| Including effect of type 2 diabetes onset age^†^ |  |  |  |
| PRS (per SD increase) | 1.78 | 1.21-2.69 | 4.6e-03 |
| Men | 2.85 | 1.22-6.80 | 1.6e-02 |
| Age (per year increase) | 1.06 | 1.00-1.11 | 3.0e-02 |
| Royal Victoria Hospital | 0.36 | 0.16-0.80 | 1.3e-02 |
| 51-60 | 0.88 | 0.37-2.07 | 0.77 |
| >60 | 0.82 | 0.30-2.16 | 0.68 |

^*^: Relative to type 2 diabetes duration no longer than 10 years

^†^: Relative to type 2 diabetes onset age no older than 50

^^

**Supplemental Figure S1.** Ethnicity decomposition of local population. 162,811 representative LD-pruned SNPs were used to examine similarity of each individual with the Eastern Asian (EAS), African (AFR) and European (EUR) populations from the 1,000 Genomes Project (G1000 cohort). Samples residing in the shaded area were regarded as European descendants. Samples residing in the middle of two super populations may have an unlisted ancestry or a mixed ancestry, and were thus not regarded as European descendants Samples recruited at the two different hospitals were shaped differently. AFR: the African super population; EAS: The East Asian super population; EUR: The European super population; MCCD: The McGill Cardiac Complications in Diabetes cohort.

**Supplemental Figure S2.** Sex-specific associations of CHD PRS with CHD and clinical risk factors among individuals with type 2 diabetes. (a) Female-specific associations based on 8,092 women in the diabetic UK Biobank cohort and 65 women of a European ancestry in the MCCD cohort. (b) Male-specific associations based on 13,010 men in the diabetic UK Biobank cohort and 245 men of a European ancestry in the MCCD cohort. All results were based on logistic regression models as presented in Figure 1 without adjusting for sex.

b

a

**Supplemental Figure S3.** Meta-analyses of ORs based on (a) logistic regression models using multivessel stenosis as the outcome and (b) ordinal logistic regression models using the number of atherosclerotic lesions as the outcome. Regressions were performed separately by hospital on samples with a 0putative European ancestry in the MCCD cohort. Estimated ORs are represented by squares where the area is proportional to the corresponding sample size at each hospital. Meta-analytic ORs are represented by diamonds. For all ORs, the corresponding CIs are indicated inside brackets. JGH: The Jewish General Hospital; RVH: The Royal Victoria Hospital.
